# Rapid Induction of Protective Immunity against Pneumonic Plague by *Yersinia pestis* Polymeric F1 and LcrV Antigens

**DOI:** 10.3390/vaccines11030581

**Published:** 2023-03-02

**Authors:** Moshe Aftalion, Avital Tidhar, Yaron Vagima, David Gur, Ayelet Zauberman, Tzvi Holtzman, Arik Makovitzki, Theodor Chitlaru, Emanuelle Mamroud, Yinon Levy

**Affiliations:** 1 Department of Biochemistry and Molecular Genetics, Israel Institute for Biological Research, P.O. Box 19, Ness Ziona 74100, Israel; 2 Department of Biotechnology, Israel Institute for Biological Research, P.O. Box 19, Ness Ziona 74100, Israel

**Keywords:** vaccination, pneumonic plague, rapid protection, polymeric F1, monomeric F1, LcrV, mouse model

## Abstract

In a recent study, we demonstrated that vaccination with the polymeric F1 capsule antigen of the plague pathogen *Yersinia pestis* led to the rapid induction of a protective humoral immune response via the pivotal activation of innate-like B1b cells. Conversely, the monomeric version of F1 failed to promptly protect vaccinated animals in this model of the bubonic plague. In this study, we examined the ability of F1 to confer the rapid onset of protective immunity in the more challenging mouse model of the pneumonic plague. Vaccination with one dose of F1 adsorbed on aluminum hydroxide elicited effective protection against subsequent lethal intranasal exposure to a fully virulent *Y. pestis* strain within a week. Interestingly, the addition of the LcrV antigen shortened the time required for achieving such rapid protective immunity to 4–5 days after vaccination. As found previously, the polymeric structure of F1 was essential in affording the accelerated protective response observed by covaccination with LcrV. Finally, in a longevity study, a single vaccination with polymeric F1 induced a higher and more uniform humoral response than a similar vaccination with monomeric F1. However, in this setting, the dominant contribution of LcrV to long-lasting immunity against a lethal pulmonary challenge was reiterated.

## 1. Introduction

The Gram-negative bacterium *Yersinia pestis* constitutes the etiologic agent of bubonic and pneumonic plague. Hundreds of millions have succumbed due to the lethal nature of infections inflicted by this devastating pathogen in three major pandemics and in numerous local outbreaks recorded in the past two millennia [1,2]. Due to its high infectivity and lethality, *Y. pestis* is classified as a potential bioterror agent (https://www.cdc.gov/plague/bioterrorism/index.html, accessed on 9 July 2021). Since the discovery and extensive use of antibiotics, the prevalence and mortality caused by the plague has sharply diminished [3,4]. Nevertheless, antibiotic therapy against the plague has limitations; to be effective, the treatment must be administered shortly after the onset of symptoms that, in the beginning, are similar to those of a common upper respiratory infection and hence often misdiagnosed. Any delay in the administration of antibiotics specific for plague therapy may impair their effectiveness and result in significant mortality among treated individuals [5]. The recent isolation of virulent *Y. pestis* strains resistant to multiple antibiotics, including those recommended for therapy, and the concern that such altered phenotypes may be maliciously introduced into virulent strains for biological warfare or bioterrorism necessitates the development of additional countermeasures [6,7,8]. Indeed, the recent application of second-line antibiotics, additional therapeutic modalities, and the combination of antibiotic and phage therapy shows promising results in the mouse model of the pneumonic plague [9,10].

We have recently demonstrated using the mouse model of the bubonic plague that administration of the F1 capsular antigen of *Y. pestis* elicits protective immunity within days and, therefore, can serve as a therapeutic intervention for the prevention of the disease [11,12]. This rapid onset of protection involved rapid activation of innate-like B1b cells that reside in the peritoneal and pleural cavities, resulting in the production of circulatory anti-F1 IgM and IgG antibodies [11,12]. The polymeric structure of F1 was essential for the rapid induction of anti-plague immunity, as demonstrated by the observation that, contrary to polymeric F1, engineered monomeric circular permutated F1 protein (monoF1) failed to associate with peritoneal B1b cells immediately after immunization, and accordingly, the production of anti-F1 antibodies was delayed [12]. In this study, using a mouse model of intranasal *Y. pestis* infection, we examined the ability of the F1 antigen to provide rapid protective immunity against pneumonic plague, which represents the most severe manifestation of the disease. This study establishes that, as in the case of bubonic plague immunity, the polymeric nature of F1 was essential for rapid as well as long-lasting immunity following vaccination with a single dose of the antigen. Since F1 and LcrV antigens represented the most likely candidate anti-plague subunit vaccines and were shown to elicit rapid protective immunity in a mouse model of the pneumonic plague [13], we extended this study by addressing the possibility of further expediting mounting the protection against pneumonic plague by a combined F1 with the LcrV formulation.

## 2. Materials and Methods

### 2.1. Ethics Statement

This study was carried out in strict accordance with the recommendations for the Care and Use of Laboratory Animals of the National Institute of Health. All animal experiments were performed in accordance with Israeli law and were approved by the Ethics Committee for animal experiments at the Israel Institute for Biological Research (Permit Numbers: IACUC-IIBR M-50-18, IACUC-IIBR M-36-19, IACUC-IIBR M-64-19, IACUC-IIBR M-73-20). Following the intranasal challenge, the mice were monitored daily. Humane endpoints were used in our survival studies. Mice showing loss of the righting reflex were euthanized by cervical dislocation.

### 2.2. Animals

Six- to eight-week-old inbred C67BL/6 female mice were purchased from Envigo (Jerusalem, Israel). Male and female CD-1 of similar age were purchased from Charles River (UK).

### 2.3. Animal Immunization with Purified Antigens

PolyF1: The polymeric F1 protein was purified from the culture supernatant of *E. coli* cells expressing the *caf1* operon of the *Y. pestis* Kimberley53 strain, as previously described in [14]. The molecular weight of purified F1 was determined to be higher than 1000 kDa by size-exclusion chromatography using a Superdex 200 column (Cytiva, MA, USA).

MonoF1: The plasmid pAH34L coding for the monomeric F1 (cpCaf1) was kindly obtained from Dr. Daniel Peters and Prof. Jeremy H. Lakey, Newcastle University, UK [15]. pAH34L was introduced into Lemo21 (DE3) *E. coli* purchased from New England Biolabs (Ipswich, MA, USA). Randomly selected transformants were grown at 37 °C and 220 rpm in Terrific broth supplemented with ampicillin (200 µg/mL). Expression was induced mid-log by the addition of iso-propyl β-thiogalactopyranoside (IPTG; 1 mM), and the cultures were subsequently incubated at 18 °C and 220 rpm for an additional 19 h. Bacterial pellets were weighed (wet weight) and solubilized with CelLytic™ B cell lysis reagent according to the manufacturer’s instructions (Merck-Sigma, Rehovot, Israel). The recombinant Tol3–cpCaf1 fusion protein was purified by facilitated pressure liquid chromatography (PFLC) on a HisTrap™ HP column according to the manufacturer’s guidelines (Cytiva, MA, USA). The Tol3 moiety was separated from cpCaf1 by cleavage with thrombin (Merck-Sigma, Rehovot, Israel) and reabsorption of the former on a regenerated HisTrap™ HP column. The cpCaf1 (monoF1) moiety was collected from the flow-through fraction and extensively dialyzed against 1× PBS. Endotoxins were removed by the Triton X-114 phase separation method, as described previously in [16]. Protein quantity was measured using the bincinchoninic acid method (Pierce™ BCA Protein Assay Kit, Thermo). The purity of the monomeric F1 was verified by SDS-PAGE followed by Coomassie blue staining and by Western blot analysis using rabbit anti-F1 antibodies, as previously described in [11].

LcrV: The full-length LcrV coding sequence from the virulent *Y. pestis* Kimberley53 strain was cloned into the pGEX expression vector (GE Healthcare, Chicago, IL, USA) and introduced into *E. coli* cells. The LcrV coding sequence was found to be identical to LcrV of the *Y. pestis* CO92 strain. Recombinant LcrV was produced and purified as described by Leary et al. [17]. Endotoxins were removed from purified LcrV as described above.

A total of 20 to 160 µg of polyF1 and LcrV or 80 µg of monoF1 were adsorbed on 2% aluminum-hydroxide (AlOH) gel (Brennentag Biosector, Denmark) to a final concentration of 0.36%. Individual mice were immunized subcutaneously using a constant volume of 200 µL administered to the lower back.

### 2.4. Characterization of the Serological Response

Mice were tail-bled, and individual IgG titers generated against F1 and LcrV in the serum were determined by ELISA, as previously reported in [11]. IgG1 and IgG3 titers were measured using isotype-specific alkaline phosphatase-conjugated goat anti-mouse antibodies (Jackson Laboratories, Farmington, CT, USA).

Bronchoalveolar lavage (BAL): Mice were anesthetized by a solution of ketamine/xylazine, and the trachea was exposed, taking extra care not to cause any bleeding. A small incision was made, and a 26-gauge flexible cannula (BD Neoflon™) was inserted and secured by a surgical suture. The lungs were washed with 1× PBS using a 1 mL luer-lock syringe that was connected to the secured cannula. Lavage fluid that was contaminated with any sign of blood was discarded. The lavage fluid was cleared by centrifugation (400× *g*, 4 °C), and the supernatant was sterile-filtered prior to ELISA. Anti-F1 and anti-LcrV antibody concentrations in the BAL fluid (BALF) of vaccinated animals were determined using diluted Mab 57 (αF1) and Mab 59 (αLcrV) previously reported in [18] as standard curves included in each 96-well test plate. The limits of detection (LODs) for anti-F1 IgG and anti-LcrV antibodies in the BALF were determined to be 0.8 ng/mL and 0.15 ng/mL, respectively.

### 2.5. Mouse Infection

Intranasal (i.n.) infections were performed as described previously in [19]. Briefly, a loop full of typical *Y. pestis* Kim53 bacterial colonies was harvested and diluted in heart infusion broth (HIB, Difco) supplemented with 0.2% xylose and 2.5 mM CaCl_2_ (Sigma-Aldrich, St. Louis, MO, USA) to an OD_660_ of 0.01 and grown for 22 h at 28 °C and 100 rpm. At the end of the incubation period (OD_660_ ≈ 4), the culture was washed and diluted in saline (0.9% NaCl) to the desired infectious dose that was verified by counting colony forming units after plating and incubating on BHIA plates (48 h at 28 °C). Mice were anesthetized with a mixture of 0.5% ketamine HCl and 0.1% xylazine and then infected intranasally with 35 µL/mouse of bacterial suspension. The intranasal LD_50_ of the Kim53 strain toward C57BL/6 mice is 1100 cfu and 2100 cfu toward CD-1 mice. The LD_50_ values were calculated according to the method described by Reed and Muench [20]. Mouse morbidity and mortality were monitored on a daily basis for 21 days. At the end of this period, clearance of the pathogen was verified by plating spleen homogenates from surviving animals onto BHIA plates supplemented with 200 µg/mL streptomycin (48 h at 28 °C).

### 2.6. Characterization of the Mouse Model of the Pneumonic Plague

Intranasally infected mice were anesthetized at the indicated time point postexposure by a solution of ketamine/xylazine, and heparinized blood was drawn by heart puncture. Subsequently, the mediastinal lymph node (mdLN), lungs, and spleen were excised and mashed to a single-cell suspension onto 70 µm nylon mesh (cell strainer, Falcon, Corning, NY, USA) in 1× PBS. The number of *Y. pestis* Kim53 in the various organs and blood was determined by plating serially diluted cell suspensions onto BHIA plates supplemented with 200 µg/mL streptomycin. For histological analysis, mice were anesthetized, and their tracheas were cannulated as indicated above. A column (0.7 × 30 cm, KONTES, St. Louis, MO, USA) filled with buffered formaldehyde solution (10% final conc.) was connected to the cannula, and the lungs were “inflated” only by gravity. Fixative-filled lungs were kept in the same buffer for 14 days and then processed to form paraffin-embedded blocks. One- to three-micrometer sections were stained with standard eosin/hematoxylin (H&E) stain and with a Masson–Goldner staining kit (Merck, Rehovot, Israel) for the detection of fibrosis. In addition, lung sections were stained for *Y. pestis* using in-house rabbit anti-F1 IgG antibodies (1:10,000) and the Histostain^®^-SP kit (ZYMED^®^, Invitrogen, Waltham, MA, USA). Stained sections were imaged using an ICC3 camera coupled to an Axioscope A1 microscope (Zeiss, Germany).

### 2.7. Statistical Analysis

Significance between titers recorded in mice belonging to more than two groups was determined by nonparametric Kruskal-Wallis with Dunn’s post hoc test. The log-rank (Mantel-Cox) test was used for comparison between the survival curves obtained in the various vaccination groups. Differences were considered significant if *p* ≤ 0.05. Calculations were performed using GraphPad Prism software.

## 3. Results

In a previous study, we demonstrated that vaccination of mice with the native polymeric form of the F1 capsular antigen of *Yersinia pestis* resulted in rapid elicitation of protection against the bubonic plague. Accordingly, it was suggested that F1 administration might be considered not only as a prophylactic measure but also as a therapeutic intervention in emergency cases such as outbreaks involving naturally occurring or maliciously engineered antibiotic-resistant strains [12]. In this report, the elicitation of rapid protection was interrogated in the case of pneumonic plague using the well-established murine model consisting of exposure of mice to the pathogen by intranasal inoculation. This model was shown to preserve the pathogenicity characterizing respiratory manifestations of primary pneumonic plague (Supplementary Figure S1) [10,19,21,22,23,24].

### 3.1. Rapid Protective Immunity Afforded by F1 and LcrV in the Mouse Model of the Pneumonic Plague

**Figure 1:**
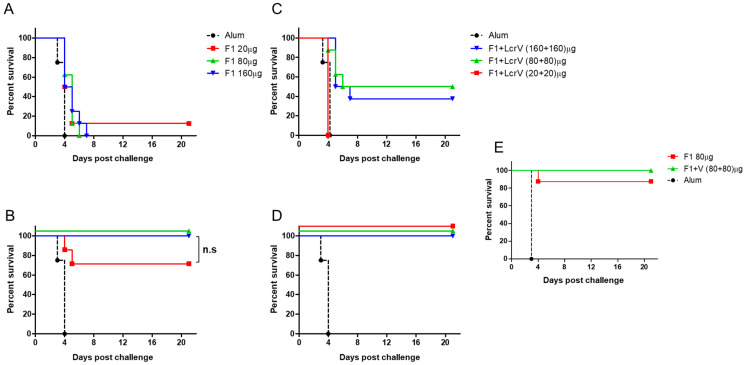
F1 and LcrV ability to induce rapid onset of immunity in a mouse model of pneumonic plague. Survival curves of C57BL/6 mice (*n* = 8) vaccinated with the indicated dose of AlOH-adsorbed polyF1 or with a mixture polyF1 and LcrV three days (**A**,**C**) or seven days (**B**,**D**), respectively, before an intranasal challenge with *Y. pestis* Kim53 (10 LD50). (**E**) Survival curves of C57BL/6 mice (*n* = 8) vaccinated either with polyF1 or with polyF1 and LcrV seven days prior to a tenfold intranasal challenge (100 LD50). Mortality of control, AlOH-vaccinated animals (*n* = 4), is marked by a dashed black line. n.s. = nonsignificant differences according to the log-rank test.

The ability of the F1 antigen to elicit rapid protection was tested by experiments in which C57BL/6 mice were vaccinated by a single administration of AlOH-adsorbed polyF1 (20, 80 and 160 µg/mouse), whereas control animals were injected only with the adjuvant. The ability of the vaccinated mice to survive lethal intranasal infection with the virulent *Y. pestis* strain Kim53 (10LD_50_ = 11,000 cfu) was evaluated three and seven days after vaccination in comparison to control animals (Figure 1). After three days, only a modest extension of the time to death and survival (of unsatisfactory statistical significance) was observed (Figure 1A). Extending the time between vaccination and challenge by only 4 more days (7 days in total) enabled the rescue of all mice vaccinated with the high doses of polyF1 (80 and 160 µg/mouse) as well as 87% of the mice vaccinated with the low dose of polyF1 (Figure 1B). The ability of the F1 antigen to induce a rapid protective immune response against the pneumonic plague encouraged us to examine whether immunization with an additional protective antigen, LcrV, would further improve the immune state of the animals by expediting the onset of protective immunity. Indeed, when the lethal *Y. pestis* challenge was carried out 3 days after the combined vaccination, the administration of the LcrV and polyF1 mixed formulation (80 µg or 160 µg of each of the antigens) was able to rescue 30–50% of the mice (Figure 1C). Most notably, extending the period between vaccination and challenge to seven days afforded full protection to all vaccinated animals at all doses of the antigens (Figure 1D). The ability of polyF1 alone or in combination with LcrV to endow rapid protective immunity against the pneumonic plague within a week was not affected by increasing the challenge dose of intranasal exposure by tenfold (Figure 1E), confirming the robustness of the protected state.

### 3.2. Kinetics of the Humoral Response Following Vaccination with F1 and LcrV

**Figure 2:**
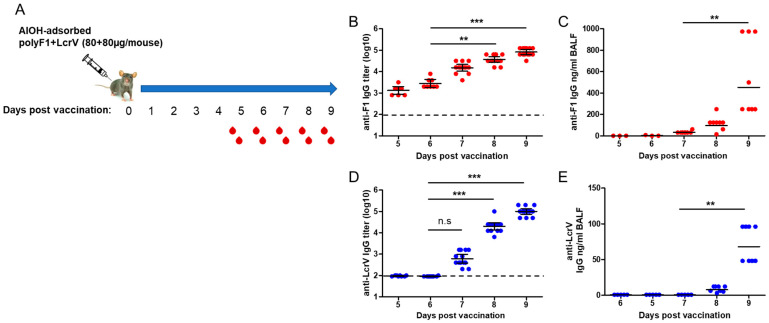
Kinetics of serological response following vaccination with polyF1 and LcrV. (**A**) Schematic description of the experiment designed to characterize the kinetics of the humoral response after a single vaccination with polyF1 and LcrV. Postvaccination days of terminal bleeding are marked below the time axis. Anti-F1 IgG endpoint titer and concentration determined in the (**B**) serum and (**C**) BALF (LOD = 0.78 ng/mL) of vaccinated mice. Anti-LcrV IgG endpoint titer and concentration measured in the (**D**) serum and (**E**) BALF (LOD = 0.15 ng/mL) of mice on the indicated days postvaccination. The horizontal dashed line marks the limit of detection in the serum. n.s.= nonsignificant, ** = *p* < 0.01, *** = *p* < 0.001.

The observation that the combined LcrV and F1 vaccine induced rapid protective immunity suggested that a humoral response was elicited shortly thereafter. To address this possibility and to determine the contribution of the humoral response against each of the antigens to protection against *Y. pestis*, the kinetics of the specific IgG antibodies generated against the two individual antigens were analyzed 5–9 days after a single-dose vaccination of C57BL/6 mice with a mixed formulation of polyF1 and LcrV (Figure 2A). As expected, on the basis of previous observations, anti-F1 IgG antibody titers (GMT = 1.35 × 10^3^) were already detectable in the sera of all vaccinated animals on Day 5 postvaccination (Figure 2B). A gradual increase in the anti-F1 IgG antibody titers was observed in the following days, reaching ~60-fold higher levels on Day 9 postvaccination (GMT = 8.26 × 10^4^, Figure 2B). Anti-F1 IgG antibodies could also be detected in the lung fluid starting from Day 7 postvaccination, reaching a concentration as high as ~1 µg/mL on Day 9 (Figure 2C). Interestingly, while the anti-LcrV humoral response lagged compared to that elicited by polyF1 (Day 7 GMT = 613, Figure 2D), a higher increase in the anti-LcrV IgG antibody levels was measured in the following days, exhibiting an overall increase of ~160-fold (GMT = 1 × 10^5^, Figure 2D). Anti-LcrV IgG antibodies could also be detected in the lung fluid of vaccinated mice starting from Day 8, albeit at a lower concentration compared to the anti-F1 levels (Figure 2E). The sharp increase in the anti-LcrV IgG titers, observed on Days 8–9 postvaccination, did not seem to involve the activation of innate-like B1b cells, as suggested by the observation that the IgG3 fraction of these antibodies was below detection compared to the anti-F1 IgG3 antibody fraction ([12] and Supplementary Figure S2). Therefore, the data indicate that LcrV could also generate a rapid humoral response providing, in combination with F1, effective protection against the pneumonic plague as early as one week after vaccination. Furthermore, the observation that anti-F1 and anti-LcrV IgG antibodies accumulated in the lungs of vaccinated animals suggests that the local response in the lungs may be important for achieving anti-pneumonic plague immunity.

### 3.3. The Importance of the F1 Polymeric Structure for the Rapid Onset of Protective Immunity

**Figure 3:**
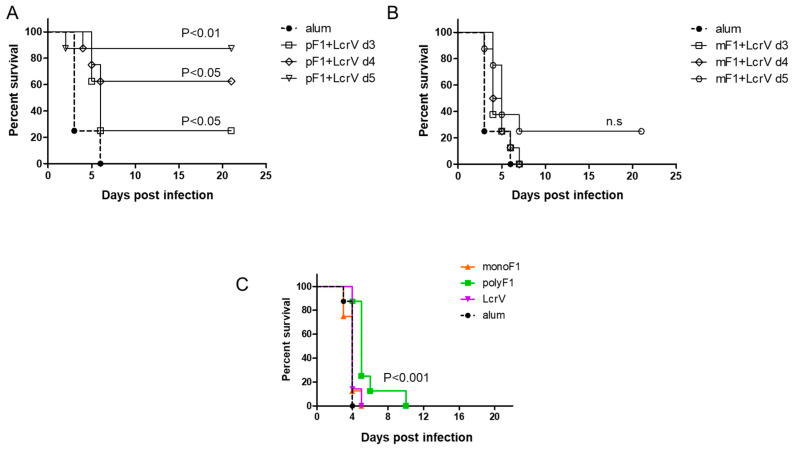
The importance of the polymeric structure of F1 in affording rapid onset of protection against pneumonic plague. (**A**) Survival curves of C57BL/6 mice (*n* = 8) vaccinated with polymeric F1 and LcrV (80 µg each/mouse) and with (**B**) monomeric F1 and LcrV (80 µg each/mouse) 3 days (open squares), 4 days (open diamonds), and 5 days (open circles) before an intranasal challenge with *Y. pestis* Kim53 (10 LD50). Mortality of control, AlOH-vaccinated animals (*n* = 4) is shown by a thick dashed line and closed circles. (**C**) Survival of the mice vaccinated with monoF1, polyF1, or LcrV (80 µg/mouse) 4 days prior to the challenge indicated above. *p* values calculated by the log-rank test toward control AlOH-vaccinated animals are shown. n.s. = nonsignificant.

We have previously published findings strongly supporting the notion that the quaternary polymeric architecture of F1 is essential for its ability to induce a rapid protective response against the bubonic plague [12]. Consequently, in this study, this issue was also addressed in the case of protection against pneumonic plague. Mice were vaccinated with monomeric or polymeric F1, in both cases entailing coadministration of LcrV (80 µg of each antigen/mouse). Three, four, and five days later, mice were challenged intranasally with a lethal dose of *Y. pestis* Kim53. The survival data depicted in Figure 3 indicated that vaccination with a mixture of polyF1 and LcrV protected 25%, 62.5%, and 87.5% of the mice, respectively (Figure 3A), from lethal challenge. In contrast, all mice vaccinated with the monoF1 and LcrV mixture succumbed to the infection except for the group vaccinated 5 days before the challenge, which exhibited a low level of 25% protection (Figure 3B). Vaccination with nonmixed formulations consisting of either polyF1, monoF1, or LcrV (80 µg/mouse) 4 days before the challenge did not protect animals. However, vaccination with polyF1 afforded a significant delay in mortality compared to all other experimental groups (*p* < 0.001; Figure 3C). Taken together, these results clearly show that the polymeric form of F1 is essential for obtaining a rapid protective response in combination with LcrV.

### 3.4. Characterization of the Long-Term Protective Immunity Induced by a Single Vaccination with F1 and LcrV

**Figure 4:**
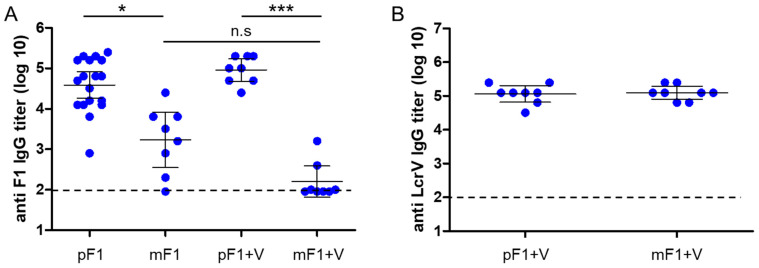
Longevity of the serological response following a single vaccination with monomeric F1 and polymeric F1 either alone or with LcrV. (**A**) Anti-F1 IgG titers measured in the sera of CD-1 female mice 180 days post a single vaccination with polymeric F1 (pF1, 80 µg/mouse), polymeric F1 + LcrV (pF1 + V, 80 µg each/mouse), monomeric F1 (mF1, 80 µg/mouse), and monomeric F1 + LcrV (mF1 + V, 80 µg each/mouse). (**B**) Anti-LcrV IgG titers that were measured in the sera of the mice as described in (**A**). The dashed horizontal line marks the limit of detection. n.s. = nonsignificant, * = *p* < 0.05, *** = *p* < 0.001.

Finally, we examined whether the classical long-term protective immunity against the pneumonic plague differs in mice vaccinated by monoF1 or polyF1. Accordingly, CD-1 mice were vaccinated once with monoF1 or polyF1 alone, as well as by administration of formulations that included combination with LcrV. The level of specific antibodies in the serum of vaccinated animals was measured six months later. The data depicted in Figure 4A show that the anti-F1 IgG titers were higher in all groups vaccinated with polyF1 than in those vaccinated with monoF1. High and consistent anti-LcrV IgG titers were measured in all mice vaccinated with LcrV (combined with either poly F1 or monoF1) (Figure 4B).

**Figure 5:**
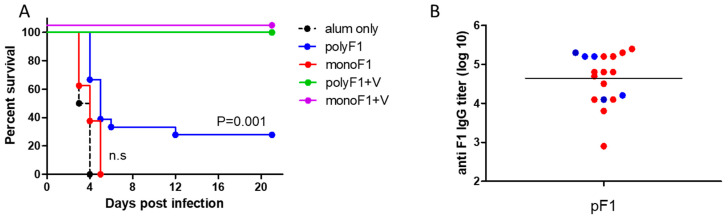
Longevity of immunity afforded by a single vaccination with monomeric F1 and polymeric F1 and LcrV in the mouse model of pneumonic plague. (**A**) Survival curves of female CD-1 mice vaccinated, as described in Figure 4, and challenged intranasally with *Y. pestis* Kim53 (100 LD50) 183 days after the administration of the vaccine. Mortality curves of control animals vaccinated only with AlOH are shown by black dashed lines. *p* values calculated by the log-rank test toward control AlOH-vaccinated animals are shown. n.s. = nonsignificant. (**B**) Correlation between the anti-F1 IgG titers measured in the serum of female mice prior to the challenge described above. Animals that survived the challenge are marked with blue symbols, and those that succumbed to the infection are marked with red symbols.

A period of 6 months postimmunization (3 days after blood withdrawal for titer determination), mice were challenged intranasally with a lethal dose consisting of 100LD_50_ of *Y. pestis* Kim53, and survival was monitored for 21 days (Figure 5). Mice vaccinated with polyF1 as a single antigen were only partially protected, whereas all mice that were vaccinated with monoF1 succumbed to the infection, similar to control naïve mice (Figure 5A). Notably, the anti-F1 IgG titers that were measured in the sera of polyF1-vaccinated animals did not exhibit a strong correlation with survival against pulmonary challenge (Figure 5B). The addition of LcrV enabled full protection for the mice that were vaccinated with monoF1 as well as for those that were vaccinated with polyF1 (Figure 5A), indicating that the anti-LcrV response enabled full manifestation of the protective value of the anti-F1 response. Similar results regarding humoral responses and survival upon pulmonary challenge were obtained in vaccinated male CD-1 mice (Supplementary Figure S3), indicating that the study is not biased by animal sex (note that the initial experiments were performed with female animals).

Taken together, these results show that F1 by itself can induce rapid onset of protection against the pneumonic plague, which depends on its natural polymeric structure. The addition of LcrV to polyF1 not only shortened the time required to achieve such rapid protection but also provided prolonged and efficacious protective immunity against the pulmonary manifestation of plague, as previously described.

## 4. Discussion

The recent isolation of virulent *Y. pestis* strains that are resistant to several antibiotics, including those recommended for therapy [8,25,26], is attributed to the search for additional countermeasures against the pneumonic plague, such as vaccines that induce rapid immunity, high public health, and clinical relevance. Vaccines, in general, are considered a preventive countermeasure against infectious diseases and are thus delivered to healthy people years in advance. Accordingly, majority of the preclinical studies examining the development of a vaccine against the plague involved prolonged vaccination regimes prior to a lethal challenge [13,27,28,29]. We have previously reported that vaccination with the natural polymeric form of the capsular F1 antigen triggers a very rapid and potent B-cell-dependent immune response that protects the animals even several hours after a lethal subcutaneous *Y. pestis* challenge [11,12]. In these studies, we have shown that a single vaccination with F1 induces the accumulation of anti-F1 antibodies (IgM and IgG) in the serum within several days via the activation of innate-like B1b cells. This subpopulation of B cells resides in the peritoneal cavity of mice and usually generates serological responses against nonproteinaceous polymeric antigens, such as LPS and polysaccharides that compose the capsule of many pathogens (pneumococci, meningococci, etc.) [30]. These rapid immune responses are characterized by T cell independence and the generation of the IgG3 murine antibody serotype. We showed that all abovementioned features also apply to the rapid protective response generated by F1. Furthermore, we showed that the polymeric nature of F1 is essential for this rapid immune response, as the monomeric F1 (generated by the circular permutation of the *caf1* gene) failed to activate B1b cells, lagged after the polymeric F1 in IgG titer kinetics, did not generate the IgG3 serotype, and failed to provide rapid onset of protection. This rapid response is accompanied by the conventional slower T-cell-dependent adaptive response against F1. Hence, we hypothesize that polymeric F1 generates two serological responses acting in parallel: one that is T-cell-independent via B1b cells and the second one involving follicular B cells in a T-cell-dependent manner [12].

In this report, the previous study was extended by addressing the capability of the F1 antigen to confer a rapid protective response in the more challenging mouse model of pneumonic plague. We show that a single administration of AlOH-adsorbed polymeric F1, seven days prior to a lethal intranasal challenge with a virulent *Y. pestis* strain, can induce an effective protective response (Figure 1). This surprising observation has almost no precedent, not only regarding the speed of the response but also in terms of the fact that the ability of the F1 antigen to confer protection in a mouse model of the pneumonic plague was only seldom addressed [31]. The observation reported here is in line with that documented by Derbise and her colleagues [32] with respect to the detection of anti-F1 antibodies shortly postvaccination. Here, we show that the addition of LcrV to polyF1 was not only advantageous in the long run, as reported previously [33,34], but also accelerated the protective immune response against *Y. pestis* intranasal challenge to only a few days (Figure 2 and Figure 5). The ability to generate rapid protection against the pneumonic plague by vaccination with F1 and LcrV strengthens similar findings previously reported by Williamson and colleagues [13], in which vaccination with F1 and LcrV provided rapid protection against aerosol challenge with *Y. pestis*. The sharp increase in the anti-LcrV IgG titers observed on Days 8 and 9 indicates that very potent immune mechanisms were activated by LcrV early after immunization. The lack of anti-LcrV IgG3 antibodies in the serum, their different kinetics and the lower levels of anti-LcrV IgG antibodies found in the lung fluid strongly suggest that discrete immune mechanisms were activated by the F1 and LcrV antigens, both of which most likely contributed to the rapid elicitation of the protective response (Figure 3C).

Similar to our previous observations in the bubonic plague mouse model, the ability to induce a rapid protective immune response against the pneumonic plague depended on the polymeric structure of the F1 antigen (Figure 3). This observation suggests that the prevalent candidate anti-plague subunit vaccine, which consists of monomeric F1 and LcrV as a fusion protein [35,36], is not expected to provide rapid onset of immunity against the pneumonic plague; however, this assumption needs to be examined experimentally. The serological response generated by LcrV, despite being slower than that of polyF1 (Figure 2), still allowed the rapid protective adaptive response that prevented the lethal effect of pneumonic plague. This relatively rapid serological response probably stemmed from the ability of the LcrV antigen to form dimers or tetramers in the solution, as has been found in previous studies [37,38] and our unpublished data. In light of our findings on the effect of the multimeric structure of F1 in expediting the generation of a humoral immune response, recombinant engineering of the LcrV protein that enables multimerization to a higher extent may further accelerate the response. In line with this assumption, it is worth noting the recent report documenting that microcrystals containing F1 and LcrV were more protective than a similar vaccine composed of these antigens in a soluble form [39]. This observation suggests that increasing the density of the antigens, which inherently occurs in the F1 polymer, may improve and possibly accelerate protective humoral responses.

In addition to its ability to induce rapid induction of immunity, in experiments addressing the longevity of the protective state, single immunization with polymeric F1 generated more stable and uniform IgG titers than that with monomeric F1 (Figure 4). However, vaccination with polyF1 alone provided only partial protection against pneumonic disease. Of note, the specific anti-F1 IgG titers measured in individual mice poorly correlated with their ability to survive the challenge (Figure 5B). Vaccination of the mice with a combination of each form of F1 together with LcrV generated higher levels of protection (Figure 5). These findings are in line with previous studies that showed that vaccination with the killed-whole cell (KWC) vaccine, which induces anti-F1 IgG but not anti-LcrV IgG titers, was ineffective in providing immunity against the pneumonic plague [13,40].

While, in the longevity study, polyF1 provided only partial late immunity, this protein demonstrated the ability to fully protect the mice shortly after the vaccination, suggesting the involvement of additional short-lived immune mechanisms that contributed to the clearance of the pathogen in the rapid immunity experiments. We have recently demonstrated that vaccination with the attenuated *Y. pestis* strain EV76 triggered a nutritional defense mechanism that abrogated the siderophore-mediated virulence mechanisms of the pathogen competing for iron ions available in the host, contributing to the rapid onset of anti-plague immunity [41]. Elucidation of the supportive short-lived mechanisms that synergize with the anti-F1 humoral immune response may assist in the development of improved vaccines against pneumonic plague as well as against other lethal infectious diseases.

## Data Availability

The data presented in this study are available upon request from the corresponding authors.

## Supplementary Materials

The following supporting information can be downloaded at: https://https://www.mdpi.com/article/10.3390/vaccines11030581/s1, Figure S1: Characterization of pneumonic plague following intranasal inoculation of *Y. pestis* Kim53. Figure S2: Isotype characterization of anti-F1 and anti-LcrV antibodies in the sera of vaccinated mice. Figure S3: Longevity of the serological response and survival of male mice following a single vaccination with monomeric F1 and polymeric F1 either alone or with LcrV.
