# Supplementary material for: Rapid Induction of Protective Immunity against Pneumonic Plague by Yersinia pestis Polymeric F1 and LcrV Antigens
Source: Vaccines (Basel). 2023 Mar 2;11(3):581. doi: 10.3390/vaccines11030581 (PMC10054699; doi:10.3390/vaccines11030581)
Supplement: Supplementary file 1 [file vaccines-11-00581-s001.zip › vaccines-2187118-supplementary.pdf]

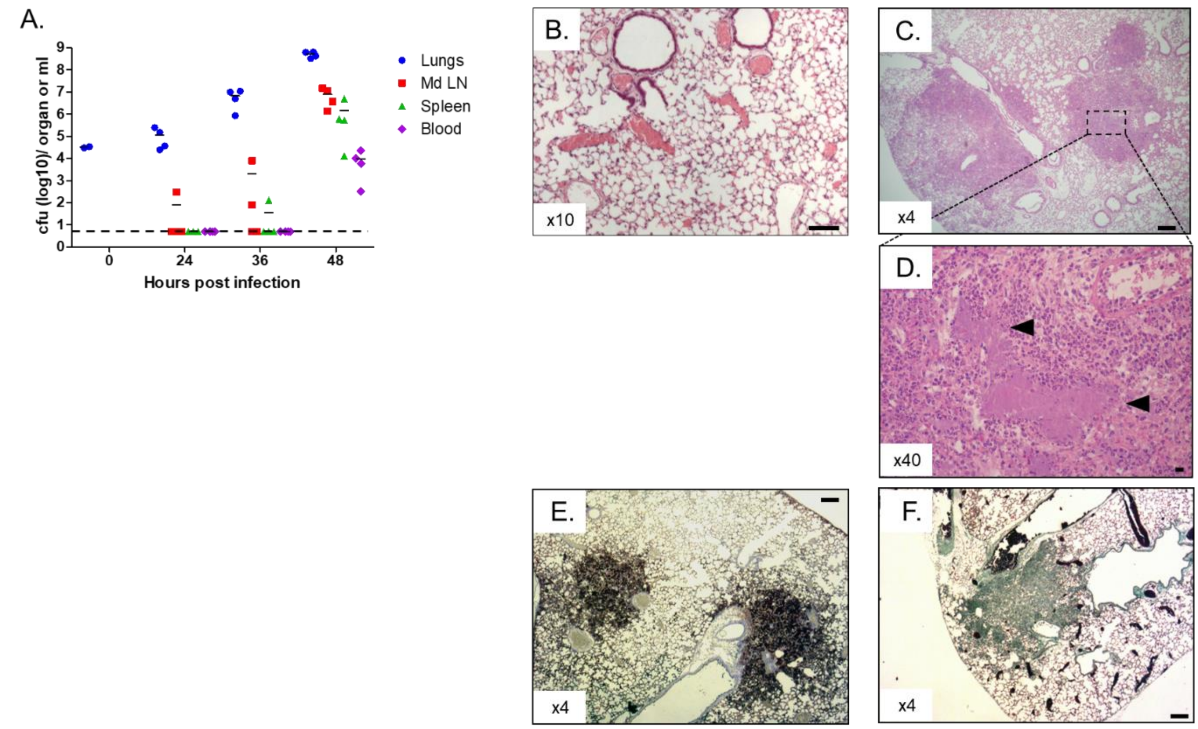

Figure S1. Characterization of pneumonic plague following intranasal inoculation of *Y. pestis* Kim53. A) Bacterial count in the lungs, mediastinal lymph node (Md LN), spleen and blood of mice at the indicated time post intranasal inoculation with 1.1×105 CFU *Y. pestis* Kim53 (100 LD50). Short horizontal lines mark the mean value of the bacterial counts in each organ and the long-dashed line marks the limit of detection that equals to 5 CFU. Eosin and hematoxylin (H&E) stain of a representative lung section obtained from (B) naïve mouse, (C) a mouse intranasally infected with *Y. pestis* Kim53 (100 LD50) and sacrificed after 48 hours, (D) the same as (C) at higher magnification of the area marked with a rectangle. Black arrows show “ponds” of *Y. pestis* surrounded by necrotizing neutrophils. E) same as (C) Immunostained with rabbit anti-F1 antibodies for the detection of *Y. pestis*. F) same as (C) stained with Trichrome Masson-Goldner staining for the detection of fibrosis. The objective lens magnification in which the lung section was photographed is marked in the bottom. Black bars mark 100µm in panels B, C, E, F and 10 µm in panel D.

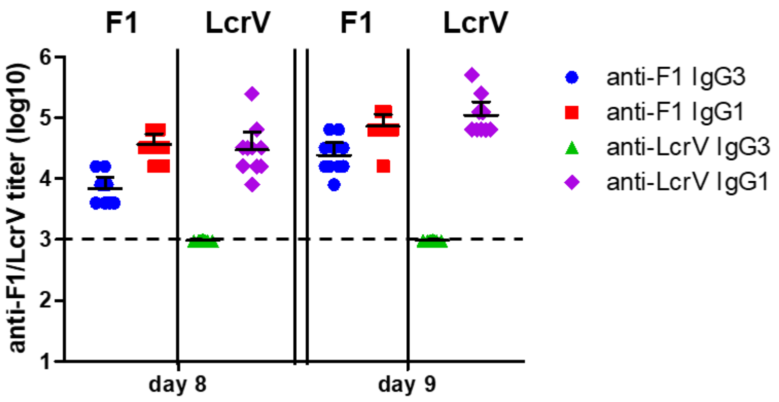

Figure S2. Isotype characterization of anti-F1 and anti-LcrV antibodies in the sera of vaccinated mice. Anti-F1 IgG3 (blue circles), anti-LcrV IgG3 (green triangles), anti-F1 IgG1 (red squares) and anti-LcrV IgG1 (purple diamonds) titers measured in the sera of C57BL/6 mice (n=10) on the indicated days post-vaccination as described in Figure 2. Dashed horizontal line marks the limit of detection.

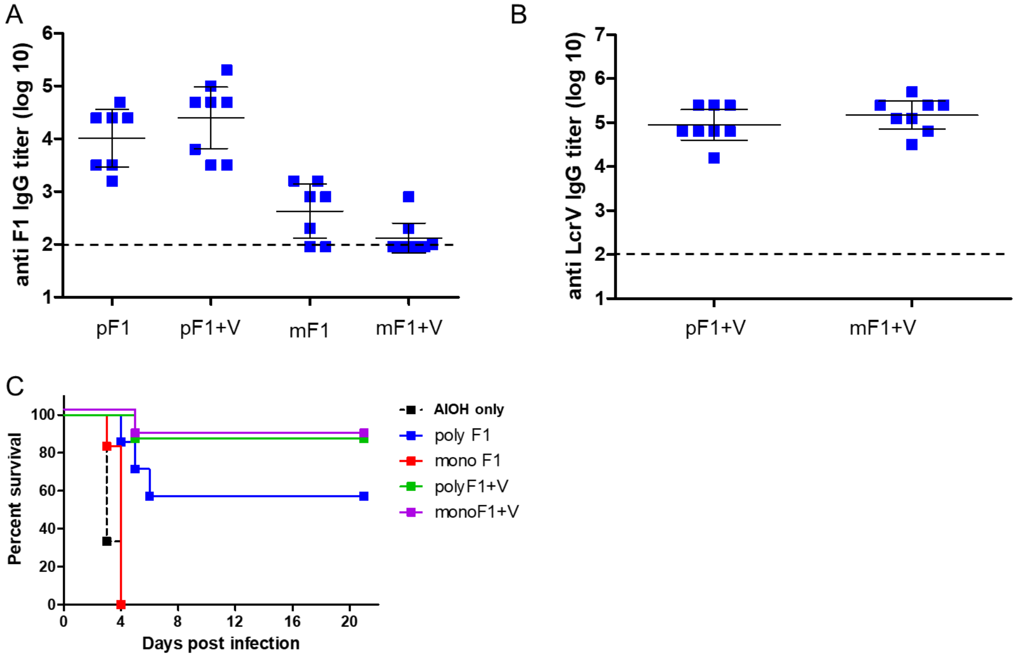

Figure S3. Longevity of the serological response and survival of male mice following a single vaccination with monomeric F1 and polymeric F1 either alone or with LcrV. A) Anti-F1 IgG titers measured in the sera of CD-1 male mice 180 days post a single vaccination either with polymeric F1 (pF1, 80µg/mouse), polymeric F1+ LcrV (pF1+V, 80µg each/mouse), monomeric F1 (mF1, 80µg/mouse) and monomeric F1+ LcrV (mF1+V, 80µg each/mouse). B) anti-LcrV IgG titers that were measured in the sera of the mice as described in (A). Dashed

horizontal line marks the limit of detection. C) Survival curves of male CD-1 mice vaccinated as described (A) and challenged intranasally with *Y. pestis* Kim53 (100 LD50) 183 days after the administration of the vaccine. Mortality curves of control animals vaccinated only with AlOH is shown by black dashed lines.
